# Bullying and cyberbullying is associated with low levels of cognitive and metacognitive learning strategies in young people

**DOI:** 10.3389/fpsyg.2025.1569400

**Published:** 2025-04-14

**Authors:** Jose Luis Solas-Martínez, Manuel J. De la Torre-Cruz, Alba Rusillo-Magdaleno, Emilio J. Martínez-López

**Affiliations:** ^1^Department of Didactics of Musical, Plastic, and Corporal Expression, University of Jaén, Jaén, Spain; ^2^Department of Psychology, University of Jaén, Jaén, Spain

**Keywords:** academic performance, adolescents, aggressors, self-regulated learning, victims

## Abstract

This study analyzed the relationship between bullying and cyberbullying, both as victims and aggressors, and the use of cognitive and metacognitive learning strategies in adolescents aged 10 to 16. A total of 1,330 Spanish students participated (48.95% boys), with an average age of 13.22 years. The Motivated Strategies for Learning Questionnaire (MSLQ) was used to assess five key learning strategies: rehearsal, elaboration, organization, critical thinking, and metacognitive self-regulation. The European Bullying Intervention Project Questionnaire (EBIP-Q) and the European Cyberbullying Intervention Project Questionnaire (ECIP-Q) were applied to evaluate levels of bullying and cyberbullying. The association between variables was analyzed through analysis of covariance (ANCOVA) and binary logistic regression analysis. The findings revealed a statistically significant negative association between bullying (traditional and cyberbullying) and the use of learning strategies for both victims and aggressors. Girls were more affected, particularly in cases of cyberbullying, where they showed lower scores in rehearsal, elaboration, and metacognitive self-regulation. In contrast, boys who were bullying aggressors scored higher in critical thinking. The risk of less frequent use of learning strategies among victims increased by 1.3 times for bullying and 2 times for cyberbullying. Similarly, this risk for aggressors rose by 1.4 times for boys and 1.8 times for girls in cases of bullying, and by 2.5 times for both genders in cases of cyberbullying. The study suggests implementing specific and cooperative actions involving students, teachers, and families to strengthen the proper use of learning strategies among victims and aggressors, especially in girls involved in cyberbullying episodes.

## 1 Introduction

Learning is influenced by multiple factors, including cognitive, emotional, and social variables (Hayat et al., 2020). Among these, peer interactions play a crucial role in shaping students’ academic experiences, with bullying and cyberbullying being particularly detrimental (Armitage, 2021; Cosma et al., 2022). Victimization and aggression in school settings can negatively impact students’ ability to process information, regulate their learning, and stay motivated (Cañas et al., 2020). Given these effects, it is essential to examine how bullying relates to the use of cognitive and metacognitive learning strategies (Aparisi et al., 2021). Cognitive and metacognitive learning strategies refer to the active mental processes students use to acquire, understand, and retain knowledge, although being aware of their strengths and limitations (Ramírez et al., 2022). Cognitive strategies for learning include rehearsal, elaboration, organization and critical thinking. On the other hand, metacognitive strategies focus on planning, monitoring and skills to control one’s own thinking, all of them determining elements in self-regulation (Pintrich et al., 1991). Both cognitive and metacognitive strategies are involved in the acquisition and consolidation of knowledge and both are key mental processes for the academic success of schoolchildren (Donker et al., 2014; Yip, 2021; Zambrano Ortega et al., 2021). In addition to enhancing self-management of learning, these strategies have been found to foster a proactive attitude toward evaluation and adaptation of study methods (Vásquez, 2021). In particular, cognitive strategies structure thinking to facilitate knowledge acquisition and retention (Neroni et al., 2019), whereas metacognitive strategies involve self-reflection and regulation of learning, promoting a growth mindset and self-efficacy (Neroni et al., 2019; Xu et al., 2022).

In line with the above, rehearsal serves as an introductory technique, activating information in working memory and proving useful for low-complexity tasks, though its effectiveness for consolidating long-term knowledge is limited, as it does not encourage integration with prior knowledge (Vásquez, 2021). Elaboration, on the other hand, involves paraphrasing, summarizing, and constructing analogies, facilitating long-term memory storage by building internal connections between new information and existing knowledge (Vásquez, 2021). Organization through grouping and outlining helps students select relevant information and establish links between new material and prior knowledge (Guo et al., 2021; Vásquez, 2021). Finally, critical thinking enables students to apply previous knowledge to novel situations, supporting problem-solving, decision-making, and evaluation according to high standards (Guo et al., 2021). In addition to these cognitive strategies, metacognitive self-regulation plays a complementary role, allowing students to plan, monitor, and regulate their cognitive processes, thereby preparing them for adaptive, self-directed learning (Guo et al., 2021; Pintrich et al., 1991; Vásquez, 2021). Importantly, both cognitive and metacognitive strategies are influenced by personal factors, including the satisfaction of psychological needs, a sense of autonomy, and academic self-efficacy (Hayat et al., 2020; Rubén and Gómez 2019). These factors can be severely compromised in contexts of stress, low self-esteem, and anxiety, often resulting from persistent bullying or cyberbullying behaviors (Aparisi et al., 2021; Menestrel, 2020; McLoughlin et al., 2020). Consequently, school bullying and cyberbullying may serve as catalysts for complex challenges with long-term negative effects on young people’s learning (McLoughlin et al., 2020; Aparisi et al., 2021). According to the Self-Regulated Learning Theory, adverse social experiences such as bullying can disrupt students’ ability to plan, monitor, and regulate their learning processes, ultimately affecting cognitive and metacognitive strategies (Li et al., 2018).

Bullying is a specific form of peer aggression that differs from general aggression in three key aspects: (1) repetition, meaning the behavior occurs over time rather than as an isolated incident, (2) intentionality, where the aggressor deliberately inflicts harm, and (3) power imbalance, which prevents the victim from defending themselves effectively (Waseem and Nickerson, 2023; UNESCO, 2019). Unlike general aggressive behaviors, which can be reactive or situational, bullying is systematic and involves a persistent dynamic of dominance and victimization (Bork-Hüffer et al., 2020). Furthermore, while general aggression may occur in response to provocation or frustration, bullying often involves strategic behaviors aimed at asserting social power over a weaker individual (Cosma et al., 2022). These distinctions are crucial in understanding the long-term psychological and educational impact of bullying. Given its repetitive nature and power imbalance, bullying involves clearly defined roles: the bully or aggressor is the individual who initiates and perpetuates the harassment, while the victim is the target of repeated aggression, often experiencing social, emotional, and academic consequences. Among these forms, traditional bullying is characterized by aggressive behavior, sometimes recurring, which manifests as a complex and harmful form of violence, occurring through direct face-to-face interactions and impacting both the physical integrity and the self-esteem of the victim (Waseem and Nickerson, 2023). According to UNESCO (2019), its prevalence varies by region, ranging from 22.8 to 48.2%. Cyberbullying, on the other hand, uses digital platforms to harass, humiliate, or threaten others, and is more common in psychological contexts, affecting girls more frequently (Vismara et al., 2022). According to Zhu et al. (2021), the prevalence of cyberbullying among young people can reach up to 57.5% in some regions. Unlike traditional bullying, cyberbullying can invade all areas of a person’s private life and give aggressors a sense of anonymity and impunity (Bork-Hüffer et al., 2020; Jones et al., 2015). Both bullying and cyberbullying victims experience a reduction in their emotional well-being and self-regulation capacity, exacerbated by anxiety and low self-esteem (Li et al., 2022; Estévez et al., 2019; Obregón-Cuesta et al., 2022). This situation can lead to difficulties in social relationships (Laninga-Wijnen et al., 2023), also affecting their ability to self-regulate their studies, concentrate, and limiting their working memory (Aparisi et al., 2021; Menestrel, 2020). However, this emotional and cognitive deterioration is not limited to victims. Aggressors also face serious negative consequences, including behavioral problems, declining academic performance, and increased tendencies toward disruptive conduct. Additionally, they often experience a significant loss of self-esteem and school motivation, which can perpetuate cycles of aggression and academic failure, further exacerbating the long-term impact of bullying (Weinreich et al., 2023). Research findings indicate that both victims and aggressors exhibit poor self-regulation, which results in a superficial approach to learning and a lower predisposition to use complex metacognitive strategies (Leiner et al., 2014; Weinreich et al., 2023). The final consequence is a decline in students’ acquisition of competencies and an increased risk of school dropout (Obregón-Cuesta et al., 2022; Weinreich et al., 2023).

In terms of gender, boys are more likely to be both victims and aggressors, frequently resorting to physical violence or threats, while girls tend to be more vulnerable to psychological harassment and are generally less involved in bullying behaviors (Cosma et al., 2022). However, some studies suggest that these differences may not always be significant, as bullying dynamics can vary depending on contextual and cultural factors (Zhu et al., 2021). Beyond bullying involvement, gender differences also extend to cognitive and metacognitive learning strategies. Girls demonstrate greater internal control, effectively applying motivation, self-assessment, and time management techniques, whereas boys tend to show lower self-regulation but excel in concentration and information processing (Ogden et al., 2023). Additionally, other biological and social factors influence students’ cognitive development and use of learning strategies. Age plays a role in students’ ability to handle complex learning and social situations (Urruticoechea et al., 2021; El Zaatari and Maalouf, 2022). Higher levels of maternal education are associated with greater parental expectations and, consequently, better academic performance (Tantoh, 2023). Moreover, families with more resources can provide their children with additional educational tools, such as tutoring and access to diverse learning materials, creating a more supportive academic environment (Fateel et al., 2021). Finally, variables such as Body Mass Index (BMI) and the amount of weekly physical activity are closely linked to executive functions (De Greeff et al., 2018; Raine et al., 2020) and influence students’ self-esteem and motivation, key aspects for academic success (Wassenaar et al., 2021). Therefore, in studying the processing of cognitive and metacognitive learning strategies, it is essential to control, as much as possible, for the potential effect of these covariates.

This study is grounded in the Social-Ecological Model of Cyberbullying (Patel and Quan-Haase, 2022), which expands on Bronfenbrenner’s ecological systems theory by integrating the digital environment as a critical factor shaping bullying experiences. This model emphasizes the interaction between individual characteristics, peer relationships, school policies, and digital media in influencing cyberbullying behaviors. From a policy perspective, this framework highlights the need for multi-tiered interventions that address not only school environments but also the influence of digital media on student behavior, recognizing that cyberbullying occurs within complex and interconnected social systems (Patel and Quan-Haase, 2022). Additionally, we draw on Self-Regulated Learning Theory (Li et al., 2018), which posits that students’ ability to monitor and regulate their learning is directly impacted by social stressors, such as victimization or aggressive behaviors, that create emotional and cognitive disruptions. In line with this, prior research has shown that bullying and cyberbullying negatively correlate with learning motivation (Aparisi et al., 2021) and academic performance (Huang, 2020; Obregón-Cuesta et al., 2022). However, despite evidence linking these phenomena to educational outcomes, the specific extent of their association with cognitive and metacognitive learning strategies remains unclear. Given that self-regulation is a key factor in academic achievement, understanding and quantifying how bullying and cyberbullying interfere with the development and application of these strategies in detail is crucial for designing effective educational interventions (Li et al., 2018). Based on this, the aim of the present study was to analyze the association between bullying and cyberbullying victimization/aggression and the cognitive and metacognitive learning strategies used by boys and girls aged 10 to 16. Given that victims and aggressors are the primary agents directly involved in these behaviors, this study focused on examining their potential impact on learning strategies. This age range was selected as it encompasses key developmental transitions, during which bullying behaviors peak and cognitive strategies become more sophisticated in response to increasing academic demands (Zhu et al., 2021; El Zaatari and Maalouf, 2022). Additionally, the study aimed to assess the level of risk posed by bullying and cyberbullying victimization/aggression in relation to lower scores in the use of cognitive and metacognitive learning strategies. Accordingly, the specific research question was: Do students who engage more frequently in bullying/cyberbullying behaviors, whether as victims or aggressors, use cognitive and metacognitive learning strategies less frequently than their peers who are not involved in such behaviors? Based on this, the hypothesis was that bullying and cyberbullying victimization/aggression would be negatively associated with the use of cognitive and metacognitive learning strategies among boys and girls aged 10–16.

## 2 Materials and method

### 2.1 Participants

A total of 1,330 Spanish children and adolescents (651 boys, 48.95%) from 7 educational centers participated in this cross-sectional quantitative study. These institutions include both primary schools and secondary schools that provided access to students aged 10 to 16 years. The selection of educational centers was based on convenience, with four centers being urban (>10,000 inhabitants) and three rural (<10,000 inhabitants), located in different provinces of southern Spain. These centers were chosen based on their availability and accessibility. Within each center, participants were selected using a random system of complete groups (intact classrooms). Anthropometric and sociodemographic characteristics are presented in Table 1. The participants were students aged between 10 and 16 years (13.22 ± 1.75 years). Boys had a higher BMI and recorded a higher level of weekly physical activity compared to girls (*p* = 0.037 and *p* < 0.001, respectively). Additionally, boys showed greater involvement in aggressive behaviors compared to girls (*p* = 0.007). On the other hand, girls scored higher in maternal education level (*p* < 0.001), academic performance (*p* = 0.007), and cognitive and metacognitive learning strategies, excluding critical thinking (all *p* < 0.001).

**Table 1:** Biometric characteristics and socio-demographic data of participants segmented by sex.

	All (*n* = 1,330)		Boys (*n* = 651)		Girls (*n* = 679)		
Variables	Mean	SD/%	Mean	SD/%	Mean	SD/%	*p*
Age (years)	13.22	1.75	13.22	1.787	13.22	1.72	0.965
Weight (kg)	52.31	13.40	54.65	14.81	50.06	11.44	<0.001
Height (m)	1.59	0.11	1.61	0.13	1.57	0.08	<0.001
BMI (kg/m^2^)	20.47	3.97	20.70	3.91	20.25	4.03	0.037
Mother’s level of mother’s (%)							
No education		4.8%		5.0%		4.7%	<0.001
Primary (EGB)		10.2%		10.6%		10.0%	
Secondary (BUP)		14.1%		11.0%		17.4%	
Professional training		13.1%		13.1%		13.3%	
University		36.6%		35.5%		38.4%	
Do not know		20.2%		24.9%		16.2%	
Weekly PA (average)	4.01	1.76	4.30	1.81	3.73	1.67	<0.001
Academic performance	6.89	1.55	6.76	1.53	7.02	1.55	0.007
Bullying victimization							
Never	198	14.9%	112	17.2%	86	12.7%	0.085
Occasionally	703	52.9%	327	50.2%	376	55.4%	
Once or twice/month	322	24.2%	156	24.0%	166	24.4%	
Once/week	83	6.2%	46	7.1%	37	5.4%	
More than once/week	24	1.8%	10	1.5%	14	2.1%	
Bullying aggression							
Never	362	27.2%	162	24.9%	200	29.5%	0.007
Occasionally	767	57.7%	371	57.0%	396	58.3%	
Once or twice/month	156	11.7%	93	14.3%	63	9.3%	
Once/week	37	2.8%	23	3.5%	14	2.1%	
More than once/week	8	0.6%	2	0.3%	6	0.9%	
Cyberbullying victimization							
Never	582	43.8%	304	46.7%	278	40.9%	0.101
Occasionally	664	49.9%	311	47.8%	353	52.0%	
Once or twice/month	61	4.6%	26	4.0%	35	5.2%	
Once/week	21	1.6%	8	1.2%	13	1.9%	
More than once/week	2	0.2%	2	0.3%	0	0.0%	
Cyberbullying aggression							
Never	755	56.8%	363	55.8%	392	57.7%	0.368
Occasionally	515	38.7%	255	39.2%	260	38.3%	
Once or twice/month	37	2.8%	23	3.5%	14	2.1%	
Once/week	23	1.7%	10	1.5%	13	1.9%	
More than once/week	0	0.0%	0	0.0%	0	0.0%	
Cognitive and metacognitive strategies							
Rehearsal	5.28	1.17	5.08	1.16	5.47	1.13	<0.001
Elaboration	4.91	1.15	4.74	1.14	5.08	1.14	<0.001
Organization	5.13	1.38	4.76	1.38	5.49	1.22	<0.001
Critical Thinking	4.63	1.19	4.58	1.16	4.67	1.22	0.139
Metacognitive Self-regulation	5.05	0.93	4.89	0.95	5.19	0.89	<0.001
Overall strategies	5.00	0.97	4.81	0.96	5.18	0.93	<0.001

Data are presented as mean for continuous variables and frequency (%) for categorical variables. BMI = Body Mass Index; SD = Standard Deviation.

### 2.2 Measures

#### 2.2.1 Dependent variables: students’ cognitive and metacognitive learning strategies

Learning strategies were assessed using the “*Motivational Strategies for Learning Questionnaire*” (Pintrich et al., 1991). This self-report instrument consists of 81 items, grouped into 15 subscales, aimed at assessing both motivational orientations toward course content and the use of various learning strategies. For the present study, only the section of the questionnaire relating to cognitive and metacognitive learning strategies, which includes 31 items, was used. These items make up a total of 5 subscales: (1) Rehearsal (e.g.*, “When I study for this class, I practice saying the material to myself over and over.”*), (2) Elaboration (e.g.*, “When reading for this class, I try to relate the material to what I already know.”*), (3) Organization (e.g.*, “When I study the readings for this course, I outline the material to help me organize my thoughts.”*), (4) Critical Thinking (e.g.*, “When a theory, interpretation, or conclusion is presented in class or in the readings, I try to decide if there is good supporting evidence.”*), and (5) Metacognitive Self-Regulation (e.g.*, “When I become confused about something I’m reading for this class, I go back and try to figure it out.”*). Responses are recorded using a Likert-type format with seven different alternatives (1 = *Completely false for me* - 7 *= Completely true for me*). Low scores indicate minimal use of the learning strategy, although high scores reflect strong engagement with that strategy. The reliability of all corresponding subscales of the MSLQ questionnaire used in this research was acceptable (Cronbach’s *α* between 0.77 and 0.89).

#### 2.2.2 Predictor/independent variables: bullying and cyberbullying

The level of bullying was assessed using the *“*European Bullying Intervention Project Questionnaire*,”* Spanish version by Ortega-Ruiz et al. (2016), which includes a total of 14 items. This questionnaire evaluates both victimization (e.g.*, “Other students have pushed or hit me on purpose”*) and aggression (e.g.*, “I have insulted a classmate to make them feel bad”*). Reliability results are acceptable (Cronbach’s α for victimization = 0.83, Cronbach’s α for aggression = 0.79). On the other hand, to assess cyberbullying, the Spanish version of the “European Cyberbullying Intervention Project Questionnaire*”* (ECIPQ; Del Rey et al., 2015) was used, which includes a total of 22 items. This instrument also differentiates between cybervictimization (e.g.*, “Someone has posted offensive comments about me online”*) and cyberaggression (e.g.*, “I have spread rumors about a classmate on social media”*). Reliability results are acceptable (α for cybervictimization = 0.87, α for cyberaggression = 0.82). Both questionnaires distinguish between two dimensions (victimization and aggression) and use a Likert-type scale with scores ranging from 1 = never to 5 = more than once a week. Low scores indicate minimal experiences or involvement in bullying or cyberbullying, although high scores reflect frequent experiences of victimization or aggression. To ensure that the measured behaviors reflect bullying rather than general aggression or conflict, both instruments assess its defining characteristics: repetition over time and power imbalance. The questionnaires explicitly frame behaviors within the last 2 months, emphasizing their recurrent nature and helping to distinguish bullying from isolated aggression. Additionally, items assess situations where the victim is at a disadvantage (Ortega-Ruiz et al., 2016), while the cyberbullying questionnaire incorporates persistent online harassment and the perceived power asymmetry due to anonymity or lack of control (Del Rey et al., 2015). Both questionnaires were administered individually and took approximately 15 min to complete. The items explore the frequency of the behaviors described over the past 2 months. To minimize response bias, the questionnaire described behaviors without explicitly labeling them as “bullying” or “cyberbullying.” While participants were fully aware of the behaviors being assessed, avoiding an oral presentation of the questions to the entire class was intended to help students feel more at ease and reduce concerns about potential consequences or retaliation, both for victims and aggressors. This approach aimed to mitigate the influence of socially sensitive or invasive questions, which can lead respondents to modify their answers due to social desirability or discomfort (Choi and Pak, 2005; Runze and van IJzendoorn, 2024).

#### 2.2.3 Confounding variables

##### 2.2.3.1 Age and mother’s education level

The age and mother’s education level of each participant were recorded through a sociodemographic data questionnaire. Age was considered a confounding variable due to its relevance in previous studies, which have shown that cognitive and emotional maturity significantly influence how individuals learn and interact with their environment (El Zaatari and Maalouf, 2022; Urruticoechea et al., 2021). It has also been established that the mother’s education level is significantly associated with academic performance and cognitive variables that strongly influence learning, such as self-regulation, attention, and working memory (Baharvand et al., 2021; Fateel et al., 2021).

##### 2.2.3.2 Body mass index and weekly physical activity level

The level of physical activity was included as a covariate, as recent research shows how physical activity influences cognitive development and academic performance in students (Li et al., 2023; Petrigna et al., 2022). It is also considered that both BMI and physical activity are related to physical and mental well-being, as well as students’ learning and self-esteem, and therefore may mediate the effectiveness of learning strategies (Bacon and Lord, 2021; Seum et al., 2022). BMI was calculated using the Quetelet formula: weight (kg) / height^2^ (m). A digital ASIMED® scale, type B, class III, and a portable SECA® 214 stadiometer (SECA Ltd., Hamburg, Germany) were used to measure weight and height. Both measurements were taken with light clothing and without shoes. Weekly physical activity level was assessed using the *PACE+ Adolescent Physical Activity Measure* (Prochaska et al., 2001). This questionnaire consists of two items that ask how many days participants accumulated 60 min of moderate or vigorous physical activity over the last 7 days and during a typical week. The final score was obtained by averaging both responses: (P1 + P2) / 2. Its reliability index was *α* = 0.79.

### 2.3 Procedure

Before data collection began, parents, teachers, and the school administration were informed about the purpose of the study. Informed consent was obtained from the parents or legal guardians. Each participant’s name was coded to ensure anonymity and confidentiality. The measurements were conducted during school hours, as arranged by the schools. The questionnaires were completed in the usual classroom environment and were supervised by researchers and classroom tutors. This study was approved by the Bioethics Committee of the University of Jaén (Spain), reference NOV.22/2.PRY. The design complies with Spanish regulations on clinical research in humans (Law 14/2007, of July 3, on Biomedical Research), the regulations on data protection of private information (Organic Law 15/1999), and the principles of the Declaration of Helsinki (2013, Brazil). It should be noted that this study was not preregistered.

### 2.4 Statistical analysis

The comparison of continuous and categorical variables between boys and girls was conducted using Student’s *t* tests and χ^2^ tests, respectively. The normality and homoscedasticity of the data were verified using the Kolmogorov–Smirnov and Levene tests, respectively. To examine whether adolescents who had never experienced bullying or cyberbullying victimization/aggression reported a higher use of cognitive and metacognitive learning strategies compared to those who had been victims or aggressors, an analysis of covariance (ANCOVA) was performed. Each cognitive and metacognitive learning strategy (rehearsal, elaboration, organization, critical thinking, and metacognitive self-regulation) was used as a dependent variable, and bullying victimization, bullying aggression, cyberbullying victimization, and cyberbullying aggression were introduced as fixed factors. Bullying and cyberbullying scores were dichotomized as follows: participants who reported never having been victims/aggressors of bullying and/or cyberbullying (questionnaire score = 1) were labeled as “Never,” whereas those who had been victims/aggressors at some point (questionnaire score > 1) were labeled as “Sometimes.” Given that many comparison groups had different sample sizes, effect size was calculated using Hedges’ *ğ*, where 0.2 = small effect, 0.5 = medium effect, and 0.8 = large effect (Martínez-López et al., 2018). The percentage difference between groups was calculated as: [(large measurement – small measurement) / small measurement] x 100. To assess the level of risk posed by bullying and cyberbullying victimization/aggression for lower values in the use of cognitive and metacognitive learning strategies, binary logistic regression was conducted. For this, the dependent variables were dichotomized using the median as a reference (Kwon et al., 2020; Lepinet et al., 2023). In each strategy, participants were classified as high ≥ median (reference group) vs. low < median (risk group). Age, BMI, mother’s education level, and weekly physical activity were used as covariates in all analyses. Missing data were handled using listwise deletion, as the proportion of cases with missing data was low (≤5%). The missing values resulted from random individual errors, such as unanswered items due to oversight or illegible responses, rather than systematic patterns. Given this minimal percentage and its randomness, its impact on the results was negligible, and alternative methods such as multiple imputation or full information maximum likelihood (FIML) were not necessary. Listwise deletion was chosen to maintain data consistency while avoiding potential biases associated with imputation techniques (Işıkoğlu and Atar, 2020). All analyses were conducted separately for boys and girls. A 95% confidence level was used for all results (*p* < 0.05). All calculations were performed using SPSS statistical software, version 25.0 for WINDOWS (SPSS Inc., Chicago).

## 3 Results

### 3.1 Analysis of covariance on bullying and cyberbullying victimization in relation to cognitive and metacognitive learning strategies

**Figure 1:**
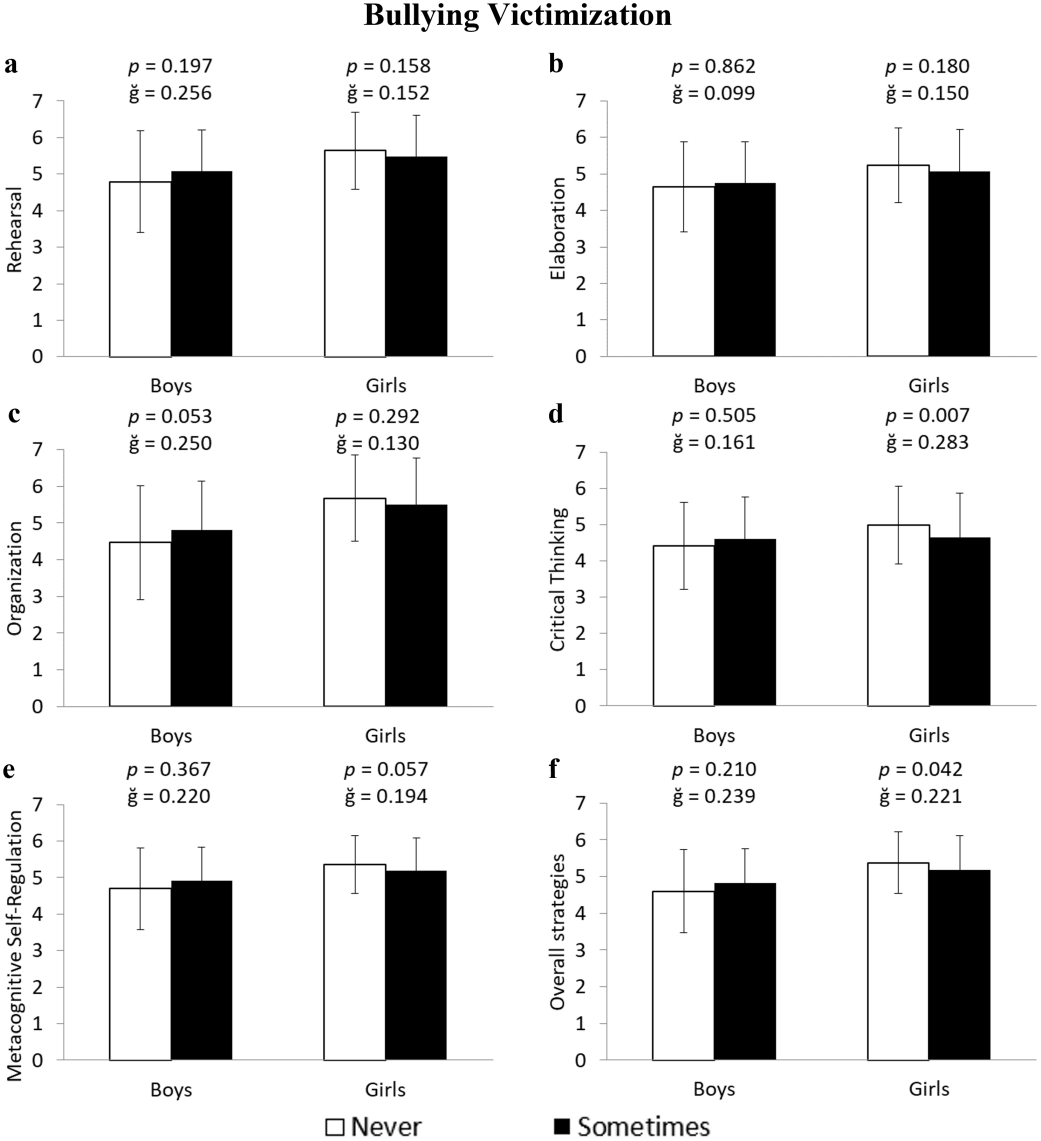
Differences between non-victims and victims of bullying in cognitive and metacognitive learning strategies in boys and girls.

Girls who were victims of bullying reported a 7.3% lower use of critical thinking strategies compared to those who had never experienced victimization (4.98 ± 1.06 vs. 4.64 ± 1.22 u.a.) *F*(1,643) = 7.325, *p* = 0.007, ğ = 0.283, 1-*β* = 0.771 (Figure 1d). No statistically significant differences were found in any other learning variables for either girls or boys in relation to bullying victimization (all *p* > 0.05; Figure 1).

**Figure 2:**
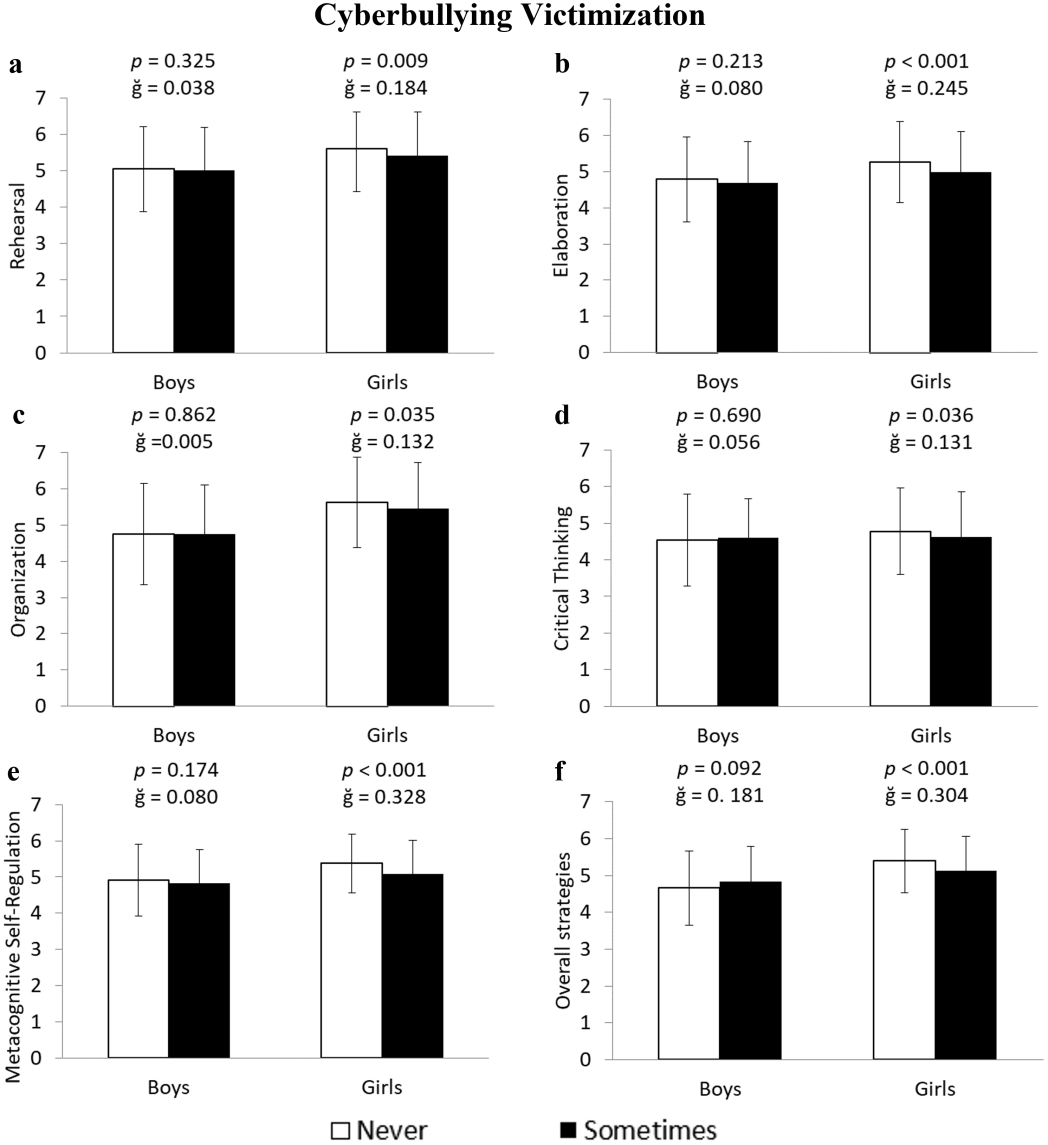
Differences between non-victims and victims of cyberbullying in cognitive and metacognitive learning strategies in boys and girls.

Meanwhile, girls who were victims of cyberbullying reported a lower use of Rehearsal: −3.8% (5.61 ± 1.01 vs. 5.40 ± 1.12 u.a.) *F*(1,643) = 6.945, *p* = 0.009, ğ = 0.184, 1-*β* = 0.749; Elaboration: −5.4% (5.25 ± 1.12 vs. 4.98 ± 1.12 u.a.) *F*(1,643) = 12.680, *p* < 0.001, ğ = 0.245, 1-*β* = 0.945; Organization: −2.9% (5.62 ± 1.24 vs. 5.46 ± 1.25 u.a.) *F*(1,643) = 4.462, *p* = 0.035, ğ = 0.132, 1-*β* = 0.559; Critical Thinking: −3.5% (4.78 ± 1.18 vs. 4.62 ± 1.22 u.a.) *F*(1,643) = 4.407, *p* = 0.036, ğ = 0.131, 1-*β* = 0.554; and Metacognitive Self-Regulation: −5.5% (5.37 ± 0.81 vs. 5.09 ± 0.92 u.a.), F(1,643) = 20.830, *p* < 0.001, 1-β = 0.995, ğ = 0.328 (Figures 2a–e, respectively). Among boys who were victims of cyberbullying, the results showed similar values to non-victims across the five learning strategies (all *p* > 0.05). An additional analysis of the average across the five learning strategies revealed that girls who were victims of bullying and cyberbullying scored 4.1 and 4.0% lower, respectively, than non-victims (5.38 ± 0.84 vs. 5.18 ± 0.93 u.a.) *F*(1,643) = 4.161, *p* = 0.042, ğ = 0.221, 1-*β* = 0.531 for bullying (Figure 1f), and (4.96 ± 0.92 vs. 4.77 ± 0.89 u.a.) *F*(1,643) = 6.761, *p* = 0.010, ğ = 0.304, 1-*β* = 0.738 for cyberbullying (Figure 2f).

### 3.2 Analysis of covariance on bullying and cyberbullying aggression in relation to cognitive and metacognitive learning strategies

**Figure 3:**
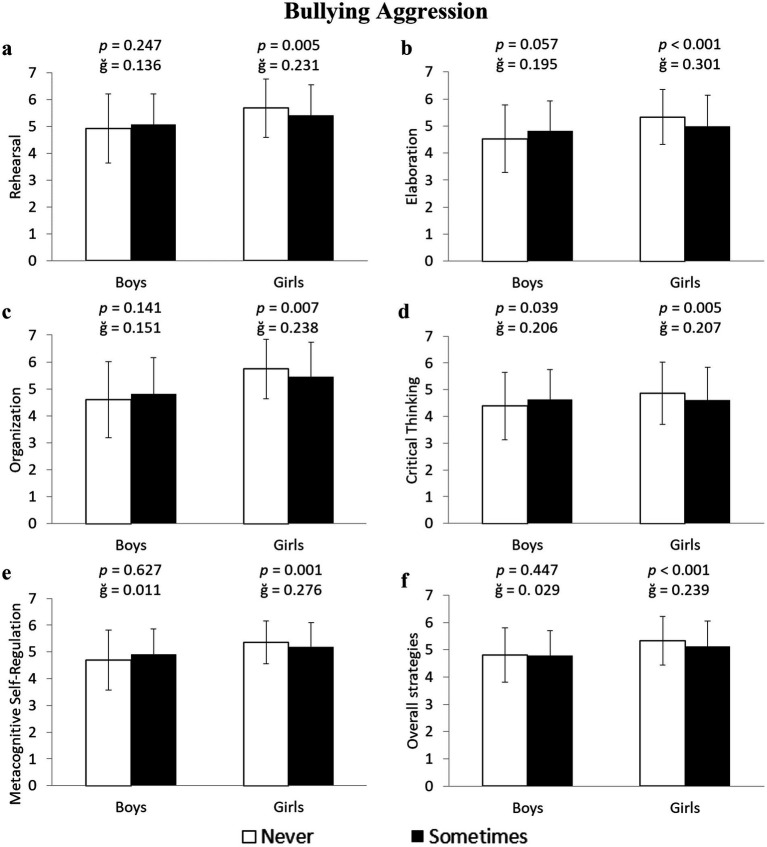
Differences between non-aggressors and aggressors in bullying in cognitive and metacognitive learning strategies in boys and girls.

Girls who were bullying aggressors scored significantly lower across all cognitive and metacognitive learning variables: Rehearsal: −3.2% (5.67 ± 1.09 vs. 5.41 ± 1.14 u.a.) *F*(1,643) = 7.999, *p* = 0.005, ğ = 0.231, 1-β = 0.806; Elaboration: −6.8% (5.33 ± 1.05 vs. 4.99 ± 1.15 u.a.) *F*(1,643) = 12.380, *p* < 0.001, ğ = 0.301, 1-β = 0.940; Organization: −5.5% (5.74 ± 1.11 vs. 5.44 ± 1.30 u.a.) *F*(1,643) = 7.362, *p* = 0.007, ğ = 0.238, 1-β = 0.773; Critical Thinking: −5.4% (4.86 ± 1.17 vs. 4.61 ± 1.22 u.a.) *F*(1,643) = 7.812, *p* = 0.005, ğ = 0.207, 1-β = 0.797; and Metacognitive Self-Regulation: −4.9% (5.38 ± 0.98 vs. 5.13 ± 0.90 u.a.), *F*(1,643) = 11.155, ğ = 0.276, *p* = 0.001, 1-β = 0.915 (Figures 3a–e, respectively). On the other hand, boys who were aggressors scored 5.5% higher in Critical Thinking compared to non-aggressors (4.63 ± 1.12 vs. 4.39 ± 1.27 u.a.) *F*(1,596) = 4.297, *p* = 0.039, ğ = 0.206, 1-β = 0.544 (Figure 3d).

**Figure 4:**
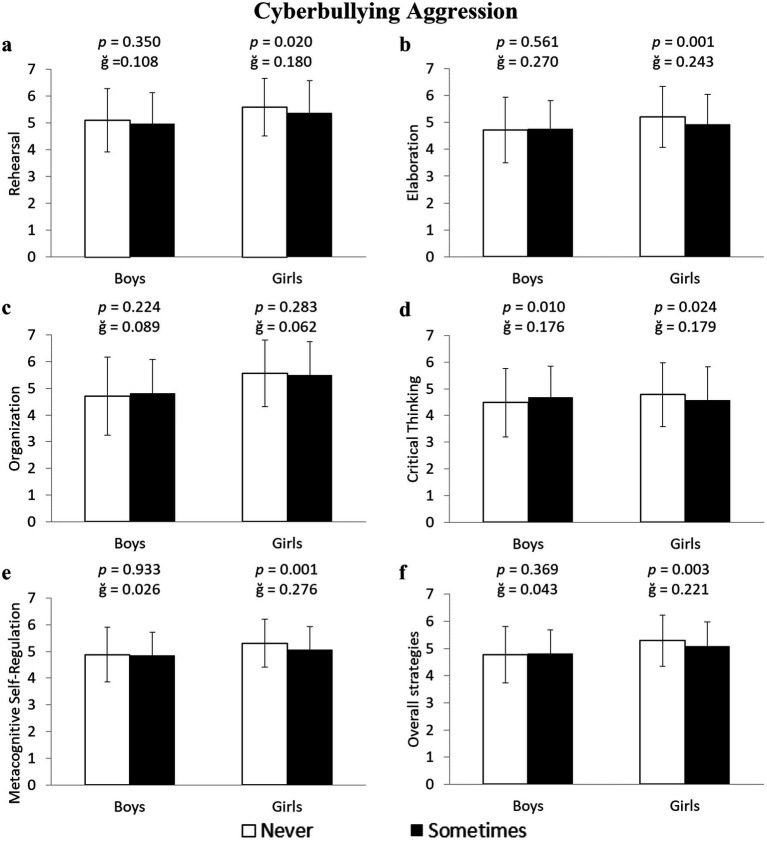
Differences between non-aggressors and aggressors in cyberbullying in cognitive and metacognitive learning strategies in boys and girls.

The results also showed that girls who were cyberbullying aggressors had lower values in four of the analyzed variables: Rehearsal: −2.6% (5.09 ± 1.18 vs. 4.96 ± 1.15 u.a.) *F*(1,643) = 5.403, *p* = 0.020, ğ = 0.108, 1-β = 0.641; Elaboration: −5.5% (5.21 ± 1.13 vs. 4.94 ± 1.12 u.a.) *F*(1,643) = 10.782, *p* = 0.001, ğ = 0.243, 1-β = 0.906; Critical Thinking: −4.8% (4.78 ± 1.20 vs. 4.56 ± 1.21 u.a.) *F*(1,643) = 5.090, *p* = 0.024, ğ = 0.179, 1-β = 0.615; and Metacognitive Self-Regulation: −4.7% (5.31 ± 0.89 vs. 5.07 ± 0.87 u.a.), *F*(1,643) = 11.897, *p* = 0.001, ğ = 0.276, 1-β = 0.931 (Figures 4a,b,d,e, respectively). Meanwhile, boys who were cyberbullying aggressors scored 4.5% higher in Critical Thinking (4.68 ± 0.97 vs. 4.48 ± 1.27 u.a.) *F*(1,596) = 6.629, *p* = 0.010, ğ = 0.176, 1-*β* = 0.729 (Figure 4d) compared to non-aggressors. No significant differences were found in the Organization strategy (*p* > 0.05; Figure 4c). An additional analysis, performed on the average of the five learning strategies, showed that girls who were bullying and cyberbullying aggressors scored 4.3 and 3.9% lower, respectively, than non-aggressors (5.33 ± 0.89 vs. 5.11 ± 0.94 u.a.) *F*(1,643) = 12.500, *p* < 0.001, ğ = 0.239, 1-β = 0.942 (Figure 3f) for bullying, and (5.29 ± 0.94 vs. 5.09 ± 0.89 u.a.) *F*(1,643) = 7.035, *p* = 0.003, ğ = 0.221, 1-β = 0.833 (Figure 4f) for cyberbullying.

### 3.3 Binary logistic regression on bullying and cyberbullying victimization in relation to cognitive and metacognitive learning strategies

Data showing the risk of exposure to bullying and cyberbullying victimization in relation to cognitive and metacognitive learning strategies are presented in Table 2. Girls who were victims of bullying were 1.26 and 1.28 times more likely, and thus at higher risk than non-victims, of having low use of Elaboration (Odds Ratio [OR] = 1.265; *p* = 0.034) and Organization (OR = 1.281; *p* = 0.027), respectively. However, no differential risk was found in boys for any of the five factors analyzed (all *p* > 0.05). On the other hand, girls who were victims of cyberbullying were more likely than non-victims to have lower scores in Rehearsal (OR = 1.724; *p* = 0.005), Elaboration (OR = 2.098; *p* < 0.001), and Metacognitive Self-Regulation (OR = 2.794; *p* < 0.001). The analysis in boys only showed a statistically significant risk for Metacognitive Self-Regulation (OR = 1.606; *p* = 0.024). An additional analysis, conducted on the average of the five learning strategies, showed that both girls and boys who were victims of bullying were more likely than non-victims to have low use of cognitive and metacognitive learning strategies (OR = 1.287; *p* = 0.025; OR = 1.266; *p* = 0.037, respectively). The data also revealed that girls and boys who were victims of cyberbullying were at higher risk of having lower scores in the use of cognitive and metacognitive learning strategies (OR = 1.905; *p* = 0.001; OR = 1.866; *p* = 0.003).

**Table 2:** Binary logistic regression for bullying and cyberbullying victimization (1 = never - 5 = more than once/week) according to categorized indicators (high vs. low) of cognitive and metacognitive learning strategies in adolescent boys and girls.

	Boys (602)					Girls (649)			
Variable		*N*	*p*	OR	95% CI	*N*	*p*	OR	95%CI
Bullying victimization									
Rehearsal	High	238		1	Referent	376		1	Referent
	Low	364	0.600	0.943	0.758–1.174	273	0.252	1.135	0.914–1.411
Elaboration	High	251		1	Referent	360		1	Referent
	Low	351	0.145	1.176	0.946–1.463	289	**0.034**	1.265	1.018–1.572
Organization	High	204		1	Referent	394		1	Referent
	Low	398	0.345	1.114	0.890–1.394	255	**0.027**	1.281	1.029–1.596
Critical Thinking	High	384		1	Referent	324		1	Referent
	Low	318	0.983	1.002	0.810–1.240	325	0.948	0.993	0.993–1.234
Metacognitive Self-Regulation	High	235		1	Referent	364		1	Referent
	Low	354	0.441	1.091	0.875–1.359	267	0.126	1.188	0.953–1.481
Overall strategies	High	262		1	Referent	392		1	Referent
	Low	340	**0.037**	1.266	1.014–1.581	257	**0.025**	1.287	1.032–1.605
Cyberbullying victimization									
Rehearsal	High	238		1	Referent	376		1	Referent
	Low	364	0.580	1.113	0.761–1.627	273	**0.005**	1.724	1.177–2.525
Elaboration	High	251		1	Referent	360		1	Referent
	Low	351	0.701	0.931	0.645–1.343	289	**<0.001**	2.098	1.401–3.140
Organization	High	204		1	Referent	394		1	Referent
	Low	398	0.064	0.705	0.488–1.020	255	0.475	1.144	0.791–1.657
Critical Thinking	High	384		1	Referent	324		1	Referent
	Low	318	0.353	0.191	0.823–1.725	325	0.414	1.169	0.803–1.701
Metacognitive Self-Regulation	High	235		1	Referent	364		1	Referent
	Low	354	**0.024**	1.606	1.063–2.426	267	**<0.001**	2.794	1.789–4.363
Overall strategies	High	262		1	Referent	392		1	Referent
	Low	340	**0.003**	1.866	1.233–2.823	257	**0.001**	1.905	1.285–2.825

OR: odds ratio, CI: confidence interval. OR was adjusted for age, BMI (body mass index), maternal education and weekly physical activity. Bold values indicate statistically significant results at *p* < 0.05.

### 3.4 Binary logistic regression on bullying and cyberbullying aggression in relation to cognitive and metacognitive learning strategies

Data showing the risk of exposure to bullying and cyberbullying aggression in relation to cognitive and metacognitive learning strategies are presented in Table 3. Girls who were bullying aggressors were 1.77, 1.78, 1.32, and 1.63 times more likely than non-aggressors to have low scores in Rehearsal (OR = 1.777; *p* < 0.001), Elaboration (OR = 1.787; *p* < 0.001), Critical Thinking (OR = 1.329; *p* = 0.049), and Metacognitive Self-Regulation (OR = 1.635; *p* = 0.001), respectively. However, in boys who were aggressors, only a statistically significant risk of low scores in metacognitive self-regulation was found (OR = 1.408; *p* = 0.024). On the other hand, girls who were cyberbullying aggressors were more likely to have low scores in Rehearsal (OR = 2.325; *p* < 0.001), Elaboration (OR = 2.571; *p* < 0.001), Critical Thinking (OR = 1.616; *p* = 0.029), and Metacognitive Self-Regulation (OR = 3.851; *p* < 0.001). Among boys who were cyberbullying aggressors, a statistically significant risk of low scores was found only for Rehearsal (OR = 1.801; *p* = 0.020) and Metacognitive Self-Regulation (OR = 2.497; *p* = 0.001). An additional analysis, conducted on the average of the five learning strategies, showed that both girls and boys who were bullying aggressors were more likely than non-aggressors to have lower use of cognitive and metacognitive learning strategies (OR = 1.768; *p* < 0.001 and OR = 1.388; *p* = 0.028, respectively). The data also revealed that, in general, girls and boys who were cyberbullying aggressors were 2.54 and 2.48 times more likely, respectively, to have lower scores than non-aggressors in the use of cognitive and metacognitive learning strategies (OR = 2.549; *p* = 0.001 and OR = 2.489; *p* < 0.001, respectively).

**Table 3:** Binary logistic regression for bullying and cyberbullying aggression (1 = never - 5 = more than once/week) according to categorized indicators (high vs. low) of cognitive and metacognitive learning strategies in adolescent boys and girls.

	Boys (602)					Girls (649)			
Variables		*N*	*p*	OR	95% CI	*N*	*p*	OR	95% CI
Bullying aggression									
Rehearsal	High	238		1	Referent	376		1	Referent
	Low	364	0.111	1.268	0.947–1.698	273	**<0.001**	1.777	1.325–2.383
Elaboration	High	251		1	Referent	360		1	Referent
	Low	351	0.913	1.015	0.773–1.334	289	**<0.001**	1.787	1.325–2.411
Organization	High	204		1	Referent	394		1	Referent
	Low	398	0.420	1.124	0.846–1.493	255	0.182	1.208	0.915–1.593
Critical thinking	High	384		1	Referent	324		1	Referent
	Low	318	0.945	1.010	0.770–1.323	325	**0.049**	1.329	1.001–1.763
Metacognitive self-regulation	High	235		1	Referent	364		1	Referent
	Low	354	**0.024**	1.408	1.045–1.897	267	**0.001**	1.635	1.207–2.215
Overall strategies	High	262		1	Referent	392		1	Referent
	Low	340	**0.028**	1.388	1.036–1.860	257	**<0.001**	1.768	1.319–2.369
Cyberbullying aggression									
Rehearsal	High	238		1	Referent	376		1	Referent
	Low	364	**0.020**	1.801	1.095–2.960	273	**<0.001**	2.325	1.475–3.664
Elaboration	High	251		1	Referent	360		1	Referent
	Low	351	0.460	1.174	0.766–1.800	289	**<0.001**	2.571	1.584–4.172
Organization	High	204		1	Referent	394		1	Referent
	Low	398	0.663	1.104	0.708–1.722	255	0.333	1.211	0.822–1.783
Critical thinking	High	284		1	Referent	324		1	Referent
	Low	318	0.178	1.344	0.874–2.066	325	**0.029**	1.616	1.049–2.489
Metacognitive self-regulation	High	235		1	Referent	364		1	Referent
	Low	354	**0.001**	2.497	1.432–4.354	267	**<0.001**	3.851	2.125–6.979
Overall strategies	High	262		1	Referent	392		1	Referent
	Low	340	**0.001**	2.549	1.499–4.332	257	**<0.001**	2.489	1.552–3.992

OR: odds ratio, CI: confidence interval. OR was adjusted for age, BMI (body mass index), maternal education and weekly physical activity. Bold values indicate statistically significant results at *p* < 0.05.

## 4 Discussion

The main objective of this study was to analyze the association between bullying and cyberbullying victimization/aggression and the use of cognitive and metacognitive learning strategies in children and adolescents aged 10–16. The results revealed that, regardless of being a victim or aggressor of bullying and/or cyberbullying, almost all bullying behaviors are negatively associated with the use of cognitive and metacognitive learning strategies, especially in girls. Table 4 summarizes the data for victims and aggressors in bullying and cyberbullying scenarios, differentiating the cognitive and metacognitive variables along with the percentage decreases/increases and associated risk increments.

**Table 4:** Extracted results of bullying and cyberbullying in youth aged 10–16 years.

Role	Type of bullying	**Boys**		**Girls**	
			Mean difference	Risk	Mean difference	Risk
Victims	Bullying	N/A	N/A	−7.3% Critical Thinking	x1.3 < Elaboration x1.3 < Organization
	Cyberbullying	N/A	x1.6 < Metacognitive self-regulation	−3.8% Rehearsal −5.4% Elaboration −2.9% Organization −3.5% Critical Thinking −5.5% Metacognitive self-regulation	x1.7 < Rehearsal x2.1 < Elaboration x2.8 < Metacognitive self-regulation
Aggressors	Bullying	+5.5% Critical Thinking	x1.4 < Metacognitive self-regulation	−3.2% Rehearsal −6.8% Elaboration −5.5% Organization −5.4% Critical Thinking −4.9% Metacognitive self-regulation	x1.8 < Rehearsal x1.8 < Elaboration x1.3 < Critical thinking x1.6 < Metacognitive self-regulation
	Cyberbullying	+4.5% Critical Thinking	x1.8 < Rehearsal x2.5 < Metacognitive self-regulation	−2.6% Rehearsal −5.5% Elaboration −4.8% Critical Thinking −4.7% Metacognitive self-regulation	x2.3 < Rehearsal x2.6 < Elaboration x1.6 < Critical Thinking x3.9 < Metacognitive self-regulation

N/A, not applicable. Data differentiated by role (victim/aggressor) and gender.

### 4.1 Associations and risk of being a victim of bullying and cyberbullying

Our data show that girls who are victims of bullying exhibit 7.3% lower use of critical thinking compared to non-victims. This confirms that persistent harassment not only compromises the mental health and emotional well-being of adolescents but also hinders the use of learning strategies and negatively impacts academic performance (Armitage, 2021; Deol and Lashai, 2022; Menestrel, 2020). We also found that when the harassment occurs through cyberbullying, victimized girls present particularly low scores in rehearsal, elaboration, organization, critical thinking, and metacognitive self-regulation. It seems that cyberbullying victims suffer a more severe impact than those of traditional bullying due to the pervasive nature of digital harassment, which generates constant stress and profoundly affects learning motivation and self-regulation (Bork-Hüffer et al., 2020; Aparisi et al., 2021). More specifically, in the analyzed girls who suffer from cyberbullying, we found that the risk of having lower engagement in elaboration and rehearsal increases by 2.1 and 1.7 times, respectively. These results support previous studies that concluded cyberbullying is associated with negative metacognitive beliefs, such as perceptions of uncontrollability and excessive responsibility, which affect cognitive confidence, particularly in memory, and lead to school avoidance behaviors (McLoughlin et al., 2022).

Among all the strategies mentioned, we found that metacognitive self-regulation is the most affected, with cyberbullying victims being twice as likely to have lower scores than non-victims. In the context of an adolescent who is being bullied, having low cognitive self-regulation can impact their learning in various ways. For example, in class, instead of paying attention to the lesson or processing information, the adolescent may be distracted by thoughts about the bullying, such as fear of future incidents or reliving past experiences (Vacca et al., 2023). This emotional distress can make it difficult to organize thoughts, keep track of tasks, or manage time effectively. Additionally, the victim may experience feelings of doubt and low self-esteem, further weakening their ability to regulate cognitive processes and fully engage in learning (Deol and Lashai, 2022; Laninga-Wijnen et al., 2023).

On the other hand, the data presented here highlight the need to study the effects of bullying on learning separately by gender. Although girls who are victims of bullying and cyberbullying experience a more severe impact on the use of cognitive strategies, boys who are victims of bullying or cyberbullying do not seem to be as negatively affected in the use of these strategies. Previous studies report greater emotional vulnerability to bullying in females, which affects their ability to organize and control the information necessary for effective learning (Menestrel, 2020). Additionally, the stress, anxiety, and low self-esteem that girls often experience in these situations interfere with their ability to manage cognitive processes such as planning and organizing learning (Hayat et al., 2020; Leiner et al., 2014). However, in boys, this weaker association could be explained by their greater exposure to physical bullying or direct aggression, leading them to activate immediate defense mechanisms, such as verbal or physical confrontations (Gomes et al., 2022). These defensive mechanisms help mitigate the impact of bullying on cognitive and metacognitive processes, partially preserving their short-term performance (Ogden et al., 2023; Cosma et al., 2022).

### 4.2 Associations and risk of being a bullying and cyberbullying aggressor

Our results revealed that girls who are bullying and cyberbullying aggressors scored significantly lower in rehearsal, elaboration, critical thinking, and metacognitive self-regulation. This implies that bullying behaviors not only affect the victims but also negatively impact the aggressors, causing difficulties in organizing and planning their studies, as well as in emotional self-regulation and social interaction (Aparisi et al., 2021; Cañas et al., 2020). We also observed that girls who are cyberbullying aggressors are at a higher risk of obtaining low scores in skills such as rehearsal, elaboration, critical thinking, and especially metacognitive self-regulation, where the risk can increase up to four times. When this is combined with the difficulty in managing negative emotions, such as anger and frustration, it likely results in a limitation in their ability to focus on tasks that require reflection and deep analysis (Yousefi et al., 2021; Estévez et al., 2019). Furthermore, whereas the anonymity of cyberbullying allows young aggressors to avoid immediate consequences, it does not mitigate the long-term emotional impact, increasing the likelihood of using learning strategies ineffectively (Vismara et al., 2022). It seems that this emotional dysregulation could be strongly linked to cognitive instability, which affects their ability to organize thoughts and plan effectively, ultimately harming their academic performance (Cosma et al., 2022; Hawley, 2015).

On the other hand, boys who are aggressors show higher use of critical thinking (5.5% for bullying and 4.5% for cyberbullying), along with high-risk values indicating a decrease in the use of cognitive strategies, particularly in metacognitive self-regulation. Although metacognitive self-regulation and critical thinking are related, they do not always develop simultaneously. Previous studies suggest that metacognitive self-regulation facilitates critical thinking, but its absence does not necessarily inhibit it (Efklides and Metallidou, 2020; Gurcay and Ferah, 2018). Among aggressors, the social context and power dynamics in bullying may encourage the use of critical thinking to justify or plan their actions (Cañas et al., 2020; Pan et al., 2023). These boys may, therefore, use critical thinking strategically to assess vulnerabilities and manipulate situations, protecting their social status, even though they do so destructively (Cosma et al., 2022; Hawley, 2015).

At this point, it is worth emphasizing some behavioral differences based on gender, which are likely influenced by the type of aggression. Girls who are aggressors tend to engage in more subtle, relational forms of aggression, such as social manipulation, exclusion, or spreading rumors, which carry a significant emotional burden (Vismara et al., 2022). This type of aggression (manipulation, exclusion, or rumor-spreading) demands a high level of emotional management, requiring girls to be constantly attuned to the social effects of their actions and the reactions of their victims. This can be cognitively exhausting and deplete their resources for using more complex learning strategies (Gomes et al., 2022; Obregón-Cuesta et al., 2022). In contrast, boys who are aggressors tend to use more direct and physical forms of aggression, which, although confrontational, do not require the same level of constant emotional monitoring. They often make impulsive decisions under pressure, limiting their ability to plan long-term, reflect on their actions, and evaluate consequences, negatively affecting their self-regulation and effective learning (Obregón-Cuesta et al., 2022). Additionally, the constant stress from physical aggression can raise cortisol levels, interfering with key cognitive processes like decision-making, planning, and concentration (James et al., 2023). Although this type of direct aggression may foster pragmatic and critical thinking skills in the short term, in the long run, it negatively impacts their ability to engage in deeper learning, which requires reflection and self-regulation (Gomes et al., 2022).

### 4.3 Recommendations to combat bullying and cyberbullying and strengthen the use of learning strategies

The findings of this study have important implications for educational policies and interventions. The Social-Ecological Model of Cyberbullying (Patel and Quan-Haase, 2022) highlights the need for multi-tiered strategies that address both school environments and digital interactions, as both influence students’ learning processes. Likewise, Self-Regulated Learning Theory (Li et al., 2018) underscores how bullying-related stress disrupts cognitive and metacognitive strategies, impairing students’ ability to plan, monitor, and regulate their learning. Addressing these challenges requires targeted interventions that strengthen self-regulation, resilience, and emotional coping skills.

Based on these considerations, prior research (Cañas et al., 2020; Efklides and Metallidou, 2020; Patel and Quan-Haase, 2022) and our data, Table 5 presents a number of study-based recommendations aimed at strengthening the use of learning strategies for both victims and aggressors of bullying and cyberbullying. These guidelines are tailored for students, teachers, and families. Gender differences and the type of aggression have been considered in their classification. It is important to note that while these recommendations are grounded in prior literature and our findings, they require further empirical validation before being considered evidence-based. These specifications do not detract from the primary focus on prevention, which seeks to avoid such consequences in the first place. In fact, to ensure the physical and mental well-being of victims, it is essential to implement preventive strategies, as well as provide awareness programs for aggressors to dissuade them from engaging in these bullying behaviors.

**Table 5:** Study-based recommendations, differentiated by gender, type of aggression (bullying or cyberbullying), and role (victim or aggressor) to maximize the impact of interventions.

Level of implementation	Type of bullying	General recommended actions	Actions to strengthen the use of learning strategies in bullying and cyberbullying victims	Actions to strengthen the use of learning strategies in aggressors of bullying and cyberbullying
Students	Bullying	Emotional support programs to improve resilience and self-esteem, along with stress management techniques to prevent bullying from affecting their well-being and academic performance.	-Boys: Organize workshops to teach them how to structure their homework and improve their planning skills. Activities such as using a daily planner to keep track of upcoming tasks will help reduce the impact of bullying on their academic performance. -Girls: Offer workshops on critical thinking and reflection on bullying situations, using case analysis exercises where they assess causes and identify bullying. This should include sessions on revision and elaboration where they learn to process and organise information effectively to improve their performance in class and their emotional resilience.	-Boys: Use their critical thinking skills to resolve conflicts in simulated situations. Programs where critical thinking is used to find non-violent solutions to conflict situations. In addition, self-regulation workshops can include emotional self-control exercises such as the ‘traffic light’ technique (pause, reflect, act). -Girls: Develop specific academic skills workshops, including exercises to improve rehearsal and organization through study techniques such as spaced repetition and concept mapping. Metacognitive self-regulation should be addressed through simulations of bullying scenarios where they practice making thoughtful decisions before acting impulsively.
	Cyberbullying	Digital literacy workshops to promote safe use of the internet and how to deal with cyberbullying, helping victims to mitigate its emotional and academic impact.	-Boys: Conduct metacognitive self-regulation workshops to teach them about healthy online time management and how to identify and deal with digital bullying. Strategies such as setting limits on social media use and learning to identify and block bullies are essential to improve their monitoring and self-efficacy. -Girls: Create self-study workshops to teach girl victims how to organize their studies. This can include elaboration techniques, such as summarizing and outlining after reading, and critical thinking, with exercises that encourage critical analysis of online messages and situations.	-Boys: Carry out reflection exercises on the impact of cyberbullying, using group dynamics to encourage critical thinking about the consequences for victims. For example, writing reflective diaries about their online behavior allows them to identify and correct aggressive patterns. -Girls: Create workshops where they run simulations that show how their actions affect victims. These simulations can include role-playing, videos or interviews with victims of cyberbullying to help them empathize. Metacognitive self-regulation should be addressed through planning and reflection activities on the use of social networks. Problem solving techniques based on real cases of cyberbullying can be used to improve reflection and elaboration.
Educators	Bullying	Teacher training to identify signs of bullying and provide a safe space where victims can express their emotions and ask for help.	Provide tools to improve the management of emotions and support the improvement of organization and critical thinking in girls. Teachers should provide an environment that promotes self-efficacy and facilitates their emotional recovery.	Train teachers in conflict mediation and teach bullies to reflect on the impact of their actions. For boys, the focus should be on developing their critical thinking skills, whereas for girls, teachers should focus on promoting respect and empathy.
	Cyberbullying	Digital literacy training for teachers to protect victims of cyberbullying and teach responsible use of social networks.	Train teachers to guide girls in the use of online time management tools and techniques, such as concept mapping, to improve their elaboration and organization skills. The use of homework tracking applications is also recommended to promote self-regulation. In the case of boys, teachers should be trained to help them set limits on the use of social networking sites in order to improve their self-regulation. Finally, include critical thinking exercises based on the analysis of bullying situations in the classroom.	Train teachers to guide bullied girls to develop their critical thinking and metacognitive self-regulation through reflection on their social media posts. The use of concept maps to help understand the impact of cyberbullying is suggested to improve rehearsal and elaboration skills. For boys, it is important to train teachers to teach them to use critical thinking to analyze patterns of online behavior, supported by reflective journals of their digital interactions.
Family	Bullying	Family programs that promote open communication, teach parents to recognize the signs of bullying and provide tools to support their children emotionally.	Involve families in emotional workshops to help them identify and manage the impact of bullying on their children, and create an environment of open communication and emotional support. In addition, families with daughters in particular should be trained to use self-regulation techniques and plan reflection sessions to encourage them to think critically about their experiences and reflect on their feelings.	Encourage critical thinking activities at home to promote peaceful conflict resolution in boys, such as problem-solving games and daily reflection routines to improve their self-regulation. For girls, it is important that families encourage reading and summarizing at home to improve their writing and organizational skills. Family reflection sessions that encourage critical thinking and respect for others are also recommended.
	**Cyberbullying**	Digital literacy sessions or training for families to monitor their children’s online activities and spot signs of cyberbullying early.	Set limits on the use of social networks and use tools such as parental control apps to improve boys’ self-regulation. For girls, it is important to teach organization and revision techniques, such as the use of digital diaries, as well as family reflection on online safety and dealing with cyberbullying.	Educate families on how to monitor and mediate their children’s online activities, promoting responsible internet use. For boys, teach them to reflect on their digital interactions and set limits on their use, in order to strengthen their self-regulation. For girls, it is important to encourage family reflection on the impact of their actions on victims, using simulations and readings that facilitate the development of critical thinking, as well as improving metacognitive processing, organization and self-regulation.

### 4.4 Limitations and strengths

This study has several methodological limitations that should be noted. Among them is its cross-sectional design, which does not allow for the establishment of causal relationships. Additionally, the sample was one of convenience, which limits its representativeness. However, the strength of the study lies in several key practices: coding techniques were applied to ensure participant anonymity and confidentiality, highly reliable measurement instruments with proven internal validity were used, and a wide range of important covariates such as age, body mass index, mother’s educational level, and weekly physical activity were considered. All of this allowed for the identification of specific results and risk levels, previously unknown, which could contribute to significant advancements in the field of education.

## 5 Conclusion

The present study concludes that the use of cognitive and metacognitive learning strategies among young people is negatively associated with both victimization and perpetration in bullying and cyberbullying. There is a more pronounced negative association for girls, with particularly severe effects observed in cases of cyberbullying. Girls who were victims of bullying were 7.3% less likely to use critical thinking than non-victims. On the other hand, girls who were victims of cyberbullying were also less likely to use the strategies of rehearsal (3.8%), elaboration (5.4%), critical thinking (3.5%) and metacognitive self-regulation (5.5%). The latter are the most affected, as girls and boys who are victims of cyberbullying are 2.8 and 1.6 times more likely, respectively, to use less them. Girls are 2.1 and 1.7 times more likely to have low use of elaboration and rehearsal strategies. In addition, aggressor girls mainly show a lower use of elaboration (6.8% for bullying and 5.5% for cyberbullying), critical thinking (5.4 and 4.8%) and metacognitive self-regulation (4.9 and 4.7%). In contrast, aggressor boys show a higher use of critical thinking (5.5% for bullying and 4.5% for cyberbullying). In terms of probability, aggressor girls’ risk of low use of learning strategies is multiplied in the factors of rehearsal (x1.8 for bullying and x2.3 for cyberbullying), elaboration (x1.8 and x2.6), critical thinking (x1.3 and x1.6) and metacognitive self-regulation (x1.6 and x3.9), whereas for boys it is only observed in the factor of metacognitive self-regulation (x1.4 and x2.5).

It is suggested to carry out specific and collaborative actions between students, teachers and families to strengthen the appropriate use of learning strategies in victims and aggressors, especially girls who are immersed in cyberbullying episodes. It is also suggested to implement clear prevention policies and to define strong consequences for aggressors in order to ensure the well-being of students and to promote an environment that facilitates effective learning.

## Data Availability

The datasets presented in this article are not readily available because the data from this study cannot be shared as it is part of a larger research project involving multiple researchers, and maintaining the confidentiality of participants is a top priority. One of the conditions for participation was that the data would be used exclusively by our research team for the specific purposes of this project. Sharing the data externally, even if anonymized, would violate the ethical commitment made with participants and could compromise the integrity of the larger research project. We adhere strictly to ethical and legal regulations, such as the General Data Protection Regulation (GDPR), to ensure the privacy and trust of all participants. Requests to access the datasets should be directed to requests for further information or specific queries regarding the study can be directed to Jose Luis Solas Martínez (jsolas@ujaen.es). Please note that, in adherence to ethical commitments and confidentiality agreements with participants, the dataset itself cannot be provided. However, we are happy to address questions or provide aggregated information where possible, ensuring the privacy and trust of all participants remain protected.
