# Supplementary material for: Bullying and cyberbullying is associated with low levels of cognitive and metacognitive learning strategies in young people
Source: Front Psychol. 2025 Apr 14;16:1569400. doi: 10.3389/fpsyg.2025.1569400 (PMC12034706; doi:10.3389/fpsyg.2025.1569400)
Supplement: Supplementary file 1 [file Supplementary_file_1.docx]

Supplementary Material

# Preliminary Regression Analyses

To enhance transparency and replicability, this document presents the preliminary regression analyses conducted on bullying and cyberbullying victimization and aggression in relation to cognitive and metacognitive learning strategies. These analyses were performed using the continuous forms of the variables before any categorization, providing additional context for the decision to dichotomize them in the final models.

Tables 1 and 2 report the results of linear regression analyses for boys and girls separately, examining the associations between bullying and cyberbullying victimization and aggression with cognitive and metacognitive learning strategies, while controlling for age, BMI, maternal education level, and weekly physical activity level. The reported coefficients include β values, standard errors (SE), and significance levels (*p*-values).

**Table 1.** Results from the Preliminary Linear Regression Analysis Associating Bullying and Cyberbullying Victimization with Cognitive and Metacognitive Learning Strategies, Controlling for Age, BMI, Maternal Education Level, and Weekly Physical Activity Level.

|  | **Boys** | | | **Girls** | | |
| --- | --- | --- | --- | --- | --- | --- |
| **Bullying victimization** | **β** | **SE** | ***p*** | **β** | **SE** | ***p*** |
| **Rehearsal** | -0.019 | 0.060 | 0.643 | -0.085 | 0.061 | 0.032 |
| **Elaboration** | -0.039 | 0.060 | 0.348 | -0.105 | 0.061 | 0.008 |
| **Organization** | -0.017 | 0.072 | 0.677 | -0.075 | 0.068 | 0.057 |
| Critical thinking | 0.059 | 0.060 | 0.145 | -0.042 | 0.065 | 0.283 |
| Metacognitive Self-regulation | -0.035 | 0.049 | 0.387 | -0.089 | 0.048 | 0.023 |
| Overall strategies | -0.011 | 0.050 | 0.781 | -0.095 | 0.050 | 0.016 |
| **Cyberbullying victimization** |  |  |  |  |  |  |
| **Rehearsal** | -0.034 | 0.105 | 0.403 | -0.145 | 0.103 | <0.001 |
| **Elaboration** | 0.007 | 0.104 | 0.866 | -0.138 | 0.103 | <0.001 |
| **Organization** | 0.069 | 0.125 | 0.093 | -0.057 | 0.114 | 0.148 |
| Critical thinking | 0.009 | 0.104 | 0.825 | -0.043 | 0.110 | 0.270 |
| Metacognitive Self-regulation | -0.080 | 0.085 | 0.048 | -0.194 | 0.080 | <0.001 |
| **Overall strategies** | <0.001 | 0.087 | 0.991 | -0.134 | 0.083 | 0.001 |

Note. SE: Standard Error

**Table 2.** Results from the Preliminary Linear Regression Analysis Associating Bullying and Cyberbullying Aggression with Cognitive and Metacognitive Learning Strategies, Controlling for Age, BMI, Maternal Education Level, and Weekly Physical Activity Level.

|  | **Boys** | | | **Girls** | | |
| --- | --- | --- | --- | --- | --- | --- |
| **Bullying aggression** | **β** | **SE** | ***p*** | **β** | **SE** | ***p*** |
| **Rehearsal** | -0.063 | 0.077 | 0.116 | -0.143 | 0.077 | <0.001 |
| **Elaboration** | -0.015 | 0.076 | 0.706 | -0.139 | 0.077 | <0.001 |
| **Organization** | 0.013 | 0.092 | 0.747 | -0.090 | 0.085 | 0.021 |
| Critical thinking | 0.055 | 0.076 | 0.168 | -0.084 | 0.082 | 0.031 |
| Metacognitive Self-regulation | -0.050 | 0.063 | 0.212 | -0.158 | 0.060 | <0.001 |
| Overall strategies | -0.012 | 0.064 | 0.770 | -0.146 | 0.062 | <0.001 |
| **Cyberbullying aggression** |  |  |  |  |  |  |
| **Rehearsal** | -0.119 | 0.118 | 0.003 | -0.188 | 0.107 | <0.001 |
| **Elaboration** | -0.016 | 0.119 | 0.701 | -0.137 | 0.108 | 0.001 |
| **Organization** | 0.030 | 0.143 | 0.473 | -0.069 | 0.120 | 0.085 |
| Critical thinking | -0.013 | 0.119 | 0.749 | -0.079 | 0.115 | 0.047 |
| Metacognitive Self-regulation | -0.092 | 0.097 | 0.022 | -0.204 | 0.083 | <0.001 |
| **Overall strategies** | -0.046 | 0.098 | 0.260 | -0.158 | 0.087 | <0.001 |

Note. SE: Standard Error
